# A Nonpeptide Oxytocin Receptor Agonist for a Durable Relief of Inflammatory Pain

**DOI:** 10.1038/s41598-020-59929-w

**Published:** 2020-02-20

**Authors:** Louis Hilfiger, Qian Zhao, Damien Kerspern, Perrine Inquimbert, Virginie Andry, Yannick Goumon, Pascal Darbon, Marcel Hibert, Alexandre Charlet

**Affiliations:** 1 Centre National de la Recherche Scientifique and University of Strasbourg, UPR3212 Institute of Cellular and Integrative Neurosciences, Strasbourg, France; 2 Laboratoire d’Innovation Thérapeutique, Faculté de Pharmacie, UMR7200 CNRS/Université de Strasbourg, Illkirch, France

**Keywords:** Receptor pharmacology, Pain

## Abstract

Oxytocin possesses several physiological and social functions, among which an important analgesic effect. For this purpose, oxytocin binds mainly to its unique receptor, both in the central nervous system and in the peripheral nociceptive terminal axon in the skin. However, despite its interesting analgesic properties and its current use in clinics to facilitate labor, oxytocin is not used in pain treatment. Indeed, it is rapidly metabolized, with a half-life in the blood circulation estimated at five minutes and in cerebrospinal fluid around twenty minutes in humans and rats. Moreover, oxytocin itself suffers from several additional drawbacks: a lack of specificity, an extremely poor oral absorption and distribution, and finally, a lack of patentability. Recently, a first non-peptide full agonist of oxytocin receptor (LIT-001) of low molecular weight has been synthesized with reported beneficial effect for social interactions after peripheral administration. In the present study, we report that a single intraperitoneal administration of LIT-001 in a rat model induces a long-lasting reduction in inflammatory pain-induced hyperalgesia symptoms, paving the way to an original drug development strategy for pain treatment.

## Introduction

Oxytocin (OT) is a 9–amino acid neuropeptide that plays an important role in several physiological and social functions. It was discovered by Sir Henry Dale for its role in lactation and parturition^1^. In the brain, OT is mainly synthesized in the paraventricular and supraoptic nuclei of the hypothalamus and released into the bloodstream by the neurons of the pituitary gland^2^. OT binds mainly to its unique receptor (OTR), a member of the G-protein coupled receptor (GPCRs) family. Its amino acid sequence was elucidated in 1953^3^ and its receptor gene was isolated in 1992^4^.

OT has been shown to induce antinociception as well as analgesia^5^. The antinociceptive and analgesic effects after intrathecal or systemic administration of OT are well-documented^6–8^. For instance, OT has a dose dependent analgesic effect in a rat model of inflammatory pain^8^, and Petersson *et al*. have shown that OT was also able to reduce the size and volume of the inflammation^7^. In addition, one study proposed that OT can also bind OTR directly in the peripheral nociceptive terminal axon in the skin^9^.

Interestingly, in nociception and pain, OT has central and peripheral targets depending on the releasing pathway: plasmatic released OT has *in vivo* antinociceptive action through reduction of C fiber excitability leading to a reduction of activity of wide dynamic range (WDR) spinal sensory neurons^10^ whereas OT released by fibers originating from PVN directly on WDR neurons inhibits sensory processing and produces analgesia in inflammatory pain model^11,12^. In these models, direct activation of parvocellular OT neuron by optogenetics, resulting in central and peripheral release of endogenous OT, also produced a significant OTR-dependent analgesia^11^.

In clinics, OT is used since many years in patients by the intravenous route for the initiation of labor and the final expulsion of the fetus^13^. It is also administered to women as a nasal spray to stimulate milk ejection. However, despite its interesting analgesic properties, OT is not used in pain treatment because it cannot efficiently penetrate the brain^14^ and is rapidly metabolized. OT half-life in the blood circulation is estimated at 5 minutes in humans and rats^15^ and around 20 minutes in rat cerebrospinal fluid (CSF)^16^. Moreover, OT suffers from several additional drawbacks: a lack of specificity, since this cyclic nonapeptide has very similar affinities for its receptor OTR, for the V1a vasopressin receptor (V1aR)^17,18^ and for the Transient Receptor Potential Vanilloid type-1 (TRPV1) of the capsaicin (EC_50_ = 0.316 μM)^19^; an extremely poor oral absorption and distribution since its high molecular weight prevents or strongly limits its absorption from the gastro intestinal tract to the blood or from the blood to the brain; and finally, a lack of patentability.

**Figure 1:**
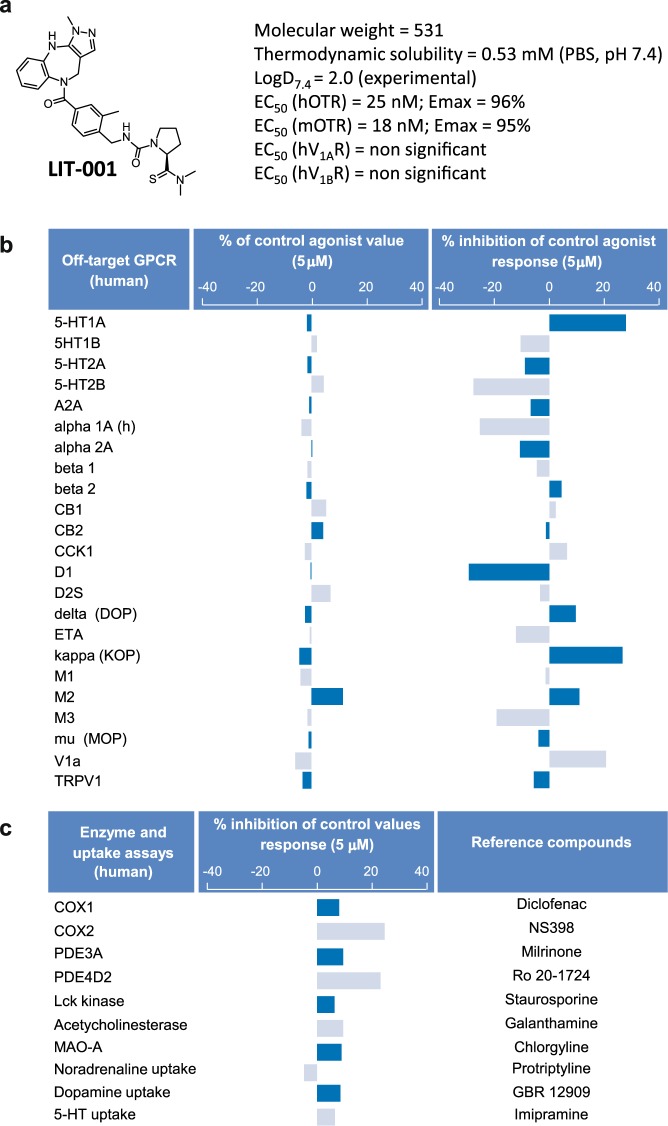
Pharmacological functional profile of LIT-001 on off-targets. (**a**) LIT-001 structure, physico-chemical properties and potency on target receptors. (**b**) *In vitro* agonist and antagonist profiles of LIT-001 on 24 off-target GPCRs. Cellular agonist and antagonist effects of LIT-001 were calculated as a % of control response to a known reference agonist for each target and cellular antagonist. Negative values are non significant in these assay setups. (**c**) Enzyme and transporter inhibition potency of LIT-001 on selected off-targets. Compound enzyme inhibition effect was calculated as a % inhibition of control enzyme activity. Compound uptake inhibition effect was calculated as a % inhibition of control uptake activity. Data are expressed as the mean value of 2 independent tests.

Recently, a first non-peptide full agonist of oxytocin (LIT-001) has been reported to improve social interactions in a mouse model of autism after peripheral administration^20^. LIT-001 is a pyrazolobenzodiazepine derivative with a non-peptide chemical structure and a low molecular weight (MW) compared to oxytocin (MW = 531 vs. 1007, respectively, Fig. 1a). Frantz *et al*. have shown that LIT-001 is a specific oxytocin receptor agonist with high affinity (EC_50_ = 25 nM and EC_50_ = 18 nM) and efficacy (Emax = 96% and 95%) for human and mouse receptors, respectively. Furthermore, the compound poorly antagonized vasopressin induced calcium release on V1aR (IC_50_ = 5900 nM) and was devoid of agonist or antagonist effect on V1bR.

In the present study, we report that a single intraperitoneal administration of LIT-001 in a rat model induces a long-lasting reduction in inflammatory pain-induced hyperalgesia symptoms, paving the way to an original drug development strategy for pain treatment.

## Results

### LIT-001, a non-peptidergic oxytocin receptor agonist

The thermodynamic water solubility (S) of LIT-001 was measured in PBS buffer at pH 7.4: S = 0.53 ± 0.03 mM (0.34 mg/mL). Its lipophilicity in the same conditions was experimentally determined: LogD_7.4_ = 2.0 ± 0.3 (Fig. 1a). The selectivity of LIT-001 (at 5 μM) has been tested on classical off-targets: 24 G-protein-coupled receptors (GPCRs), 3 transporters, 10 enzymes and 6 ion channels. No significant agonist or antagonist (Fig. 1b) activity was found at GPCRs levels or at the ionotropic TRPV1 receptor. Similarly, no significant uptake blockade is observed on noradrenaline, dopamine and serotonin transporters from rat brain synaptosomes (Fig. 1c). In addition, no enzyme inhibition activity was found on the human recombinant COX(1), PDE3A, Lck kinase, acetylcholinesterase and MAO-A from human placenta (6.4% to 9.5% inhibition, below significance) (Fig. 1c). Some inhibitory activity of COX(2) and PDE4D2 was however observed (24.6 and 23.1% at 5 μM, respectively) (Fig. 1c). On ion channels, no significant blockade activity was found on human hERG potassium, GABAA (alpha1/beta2/gamma2), Cav1.2 (L-type) calcium, Vav1.5 sodium, nAChR (alpha4/beta2) and KCNQ1/hminK potassium ion channels (Fig. S1). Finally, the lack of hERG inhibition was confirmed at two additional concentrations using patch clamp method (9.50% and 10.63% inhibition at 1 μM and 10 μM concentrations, respectively). *In vitro*, LIT-001 did not interact with CYP 2C9 and 2D6 cytochromes and weakly inhibited CYP 1A2, 2C19 and 3A4 (IC_50_ = 51, 21 and 11 μM, respectively). Interesting, tested at 1 μM, LIT-001 was very stable on human hepatocytes at 37 °C since no degradation was observed after 2 hours.

Altogether, these results indicates that LIT-001 is a very specific agonist for OTR, with limited off-targets and putative side-effects, and a long lasting (>2 h) half-life; all characteristics requested for a clinically-relevant compound.

### Ten days’ time course of long-term modifications induced by CFA subcutaneous injection

**Figure 2:**
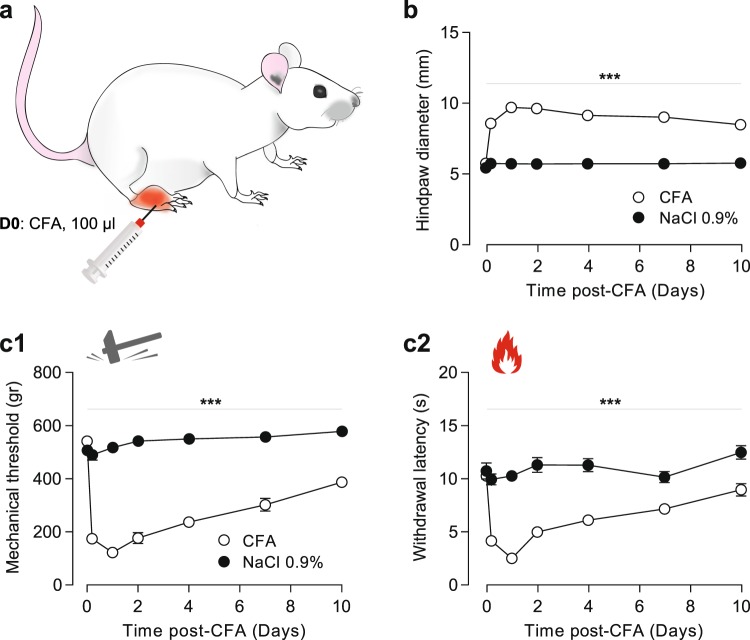
Ten days’ time course of long-term modifications induced by CFA subcutaneous injection. (**a**) Scheme of the CFA-induced inflammatory pain model (100 μl). (**b**) Time-course of the CFA-induced edema size (CFA, n = 14; NaCl, n = 18). (**c**) Time-course of the CFA-induced mechanical (**c1**) and thermal heat (**c2**) hyperalgesia (CFA, n = 14; NaCl, n = 18). Data are expressed as mean ± SEM. Asterisks indicate statistical significance (***p < 0.001) using two-way ANOVA followed by Tukey multiple comparisons post-hoc test.

Before testing the putative analgesic action of LIT-001, we started by characterizing the long-term modifications induced by a single subcutaneous injection of complete Freund adjuvant (CFA, 100 μl) in the right hindpaw (Fig. 2a). We first measured the hindpaw diameter and observed that CFA, but not NaCl 0.9%, injection induced a major edema, whose size was maximum 24 h after the injection (CFA: 9.69 ± 0.22 mm, n = 14 vs NaCl: 5.73 ± 0.14 mm, n = 11; p < 0.01) and persistent for up to 10 days (Fig. 2b). On the other hand, the mechanical CFA-induced hyperalgesia was maximum 24 h after the injection (threshold pressure CFA: 122 ± 15 g, n = 14 vs NaCl: 520 ± 14 g, n = 18; p < 0.01) and, as the edema, was persistent up to 10 days (Fig. 2c1). Similarly, a thermal heat hyperalgesia was detected for up to 10 days and maximum 24 h after the CFA injection (withdrawal latency CFA: 2.5 ± 0.23 s, n = 14 vs NaCl: 10.31 ± 0.38 s, n = 18; p < 0.01; Fig. 2c2). Interestingly, the contralateral hindpaw to the CFA-injected one did not present any mechanical nor thermal heat hypersensitivity (Fig. S2). Based on these results, we decided to test the putative analgesic properties of LIT-001 at 24 h (D1) after the CFA injection.

### Analgesic properties of LIT-001 on CFA-induced inflammatory pain model

**Figure 3:**
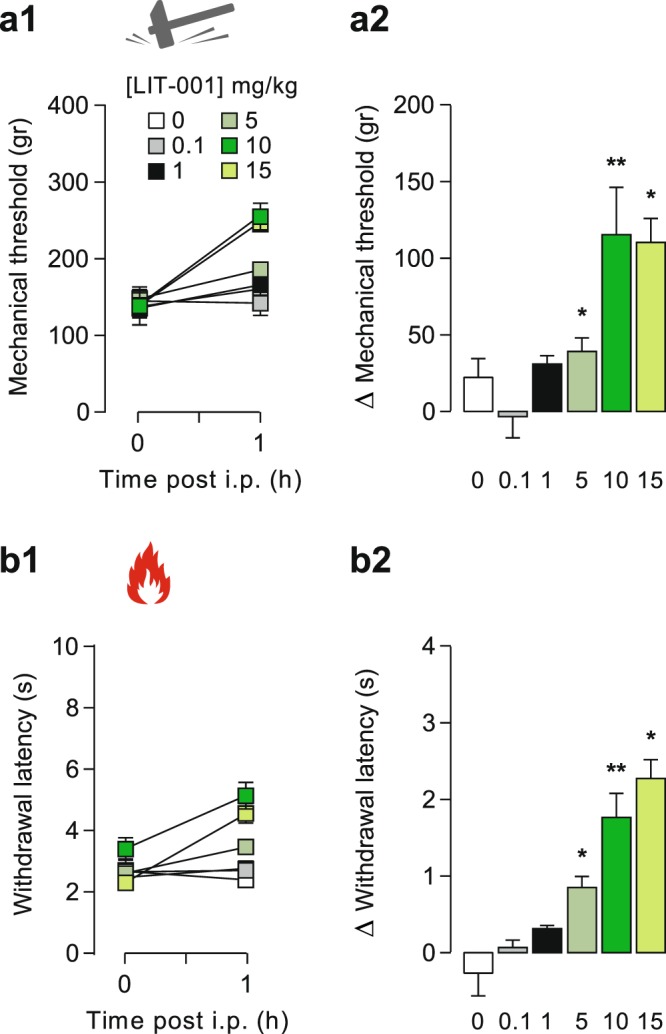
Dose-response of the analgesic properties of LIT-001 on CFA-induced inflammatory pain model. Effects of LIT-001 0.1 (n = 4), 1 (n = 6), 5 (n = 6), 10 (n = 8) and 15 mg/kg (n = 6) or its vehicule (n = 9) measured 1 hour after i.p. injection on mechanical (**a**) and thermal heat (**b**) CFA-induced hyperalgesia. Data are expressed as mean ± SEM. Asterisks indicate statistical significance (**p < 0.01; *p < 0.05) using paired Wilcoxon or T-test, depending on the data’s normal distribution.

We next aimed to test the putative analgesic properties of LIT-001 in the CFA-induced inflammatory pain model. For this purpose, we first performed a dose-response of the analgesic action of LIT-001 injected intraperitoneally (i.p.) at day 1 (D1) after the CFA injection, when the inflammatory pain symptoms were at their maximum (Fig. 3). We found a first analgesic action of i.p. LIT-001 at 5 mg/kg, an effect rising to reach a plateau at 10 mg/kg, that for both mechanical (Fig. 3a) and thermal heat (Fig. 3b) hypersensitivities. Importantly, none of the doses tested seem to exert an antinociceptive action, as measured on the contralateral hindpaw (Fig. S3).

**Figure 4:**
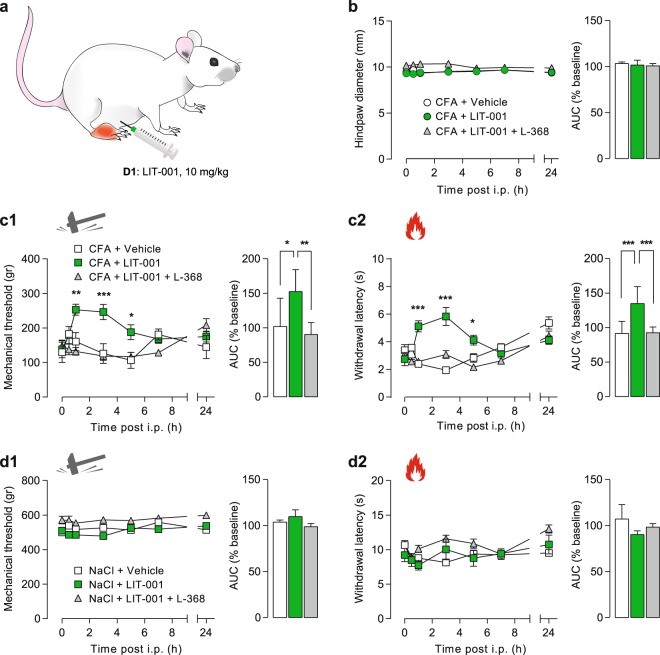
Time-course of the analgesic properties of LIT-001 on CFA-induced inflammatory pain model. (**a**) Scheme of the CFA-induced inflammatory pain model followed by i.p. LIT-001 injection. (**b**) Left, time-course of the effects of i.p. LIT-001 10 mg/kg (n = 8), its vehicule (n = 7) or co-injection with L-368,699 (n = 7), on CFA-induced edema size (CFA, n = 14; NaCl, n = 18). Right, relative-to-baseline AUC (%) of the effects. (**c**) Left, time-course of the effects of i.p. LIT-001 10 mg/kg (n = 7), its vehicule (n = 8) or co-injection with L-368,699 (n = 6) on CFA-induced mechanical (**c1**) and thermal heat (**c2**) hyperalgesia. Right, relative-to-baseline AUC (%) of the effects. (**d**) Left, time-course of the effects of i.p. LIT-001 10 mg/kg (n = 7), its vehicule (n = 8) or co-injection with L-368,699 (n = 6) on mechanical (**d1**) and thermal heat (**d2**) sensitivities of NaCl-injected hindpaw. Right, relative-to-baseline AUC (%) of the effects. Data are expressed as mean ± SEM. Asterisks indicate statistical significance (***p < 0.001; **p < 0.01; *p < 0.05) using two-way ANOVA followed by Tukey’s multiple comparisons test.

Therefore, we performed a time-course of the analgesic effect of i.p. LIT-001 (10 mg/kg) injected at day 1 (D1) after the CFA injection (Fig. 4a). For that purpose, we analyzed the hindpaw size as well as the mechanical and thermal heat hypersensitivities for 24 h (Fig. 4b–d).

We first found that i.p. LIT-001, injected alone or with a specific oxytocin receptor antagonist, L-368,699 (L-368), had no effect on the CFA-induced edema size (CFA + Vehicle, 9.44 ± 0.18 mm, n = 7; CFA + LIT-001, 9.49 ± 0.23 mm, n = 8; CFA + LIT-001 + L-368, 10.32 ± 0.17 mm, n = 7; Fig. 4b). This suggests that acute LIT-001 injection, at this stage of the CFA-induced inflammation, may have no or limited anti-inflammatory effect *per se*.

However, we revealed that i.p. LIT-001 exerts an anti-hyperalgesic action on mechanical threshold (Fig. 4c1). This effect was significant from 1 to 5 h after i.p. injection, with a maximal effect at 3 h (CFA + Vehicle, 126 ± 29 g, n = 7 vs CFA + LIT-001, 246 ± 22 g, n = 8; p < 0.001), as reflected by an increase of the area under the curve (AUC) of 152 ± 11%. We observed a similar anti-hyperalgesic action on thermal heat latency (Fig. 4c2), significant from 1 to 5 h after i.p. injection, with a maximal effect at 3 h (CFA + Vehicle, 1.94 ± 0.18 s, n = 7 vs CFA + LIT-001, 5.83 ± 0.65 s, n = 8; p < 0.001), as reflected by an increase of the AUC of 135 ± 9%. These results indicate that i.p. LIT-001 exerts a strong significant and long-lasting anti-hyperalgesic action on both mechanical and thermal heat sensitivities.

Given that LIT-001 was built as a specific agonist for OTR, we thought to validate that these anti-hyperalgesic effects were mediated by OTR activation. For this purpose, we co-injected LIT-001 with a specific OTR antagonist, L-368,699^11^. As expected, the anti-hyperalgesic action of i.p. LIT-001 was fully prevented by L-368,699, as displayed by the mechanical threshold (CFA + LIT-001 + L-368, 119 ± 12 g, n = 7) and thermal heat latency (CFA + LIT-001 + L-368, 3.08 ± 0.29 s, n = 7) and their relative AUC (90 ± 7% and 92 ± 8%, respectively) values 3 h post i.p. (Fig. 4c). These results indicate that i.p. LIT-001 likely exerts its anti-hyperalgesic action through OTR binding.

One important point in the development of a clinically-relevant anti-hyperalgesic compound is the limitation of its side effect, here the absence of anti-nociception. Noteworthy, we did not detect any alteration of contralateral hindpaw sensitivities after i.p. LIT-001, being on mechanical threshold (cCFA + LIT-001, 531 ± 25 g, n = 8; Fig. S4a1) or thermal heat latency (cCFA + LIT-001, 11 ± 0.53 s, n = 8; Fig. S4a2). Interestingly, we made similar observation on control animals, receiving NaCl 0.9% hindpaw injection and thus not presenting inflammatory pain symptoms (Figs. 4d, S4b). Here, i.p. LIT-001, injected alone or co-injected with the specific oxytocin receptor antagonist L-368,699, had no effect on mechanical (NaCl + LIT-001, 526 ± 20 g, n = 7; Figs. 4d1, S4b1) or thermal heat (NaCl + LIT-001, 8.14 ± 0.48 s, n = 7; Figs. 4d2, S4b2) hindpaw sensitivities, as reflected by the absence of increase of the AUC (Figs. 4d, S4b). These results indicate that i.p. LIT-001, as an analgesic, is only effective in case of detectable hypersensitivities.

### LIT-001 distribution and clearance in the organism

**Figure 5:**
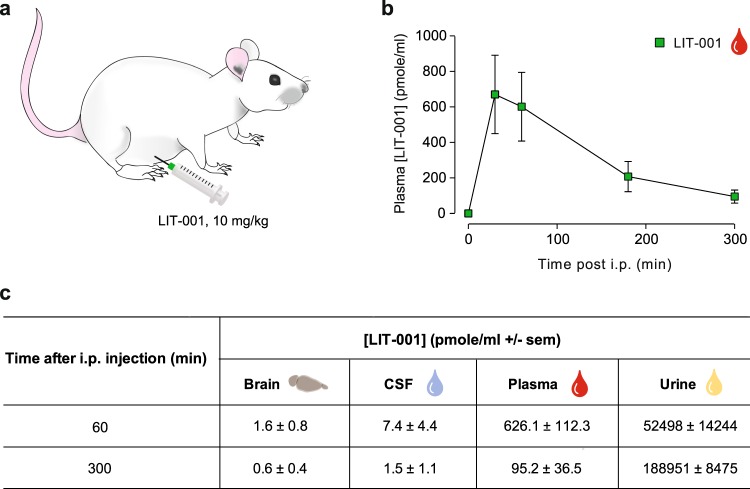
LIT-001 distribution and clearance in the organism. (**a**) Scheme displaying the i.p. injection of LIT-001, 10 mg/kg in naïve animal. (**b**) Time course of the concentrations of LIT-001 present in the plasma of rats after an i.p. injection. (**c**) Concentrations of LIT-001 present at 60 and 300 min after i.p. injection, in the brain (60, n = 6; 300, n = 5), CSF (60, n = 4; 300, n = 4), plasma (60, n = 11; 300, n = 5) and urine (60, n = 6; 300, n = 5) 60 min and 300 min after i.p. injection. Data are expressed as mean ± SEM. Data are expressed as mean ± SEM of 5 animals.

Because the anti-hyperalgesic effects of i.p. LIT-001 10 mg/kg were long-lasting, up to 5 h, while oxytocin-induced analgesia usually only last for minutes, we analyzed its distribution at key time points in plasma, CSF, brain and urine (n = 5–6, Fig. 5) and performed a quantitative dosage by comparison to a dose-response curve, using Liquid Chromatography Mass Spectrometry (LC-MS/MS; Fig. S5). Interestingly, LIT-001 concentration was found in plasma at its highest 30 min after i.p. injection (650 ± 200 pmole/ml; Fig. 5b) then slowly decreased, but was still significantly present after 300 min (5 h; 95.2 ± 36.5 pmole/ml; Fig. 5b,c). In addition, LIT-001 was found in significant amount in both the brain and CSF 60 min after i.p. injection (1.6 ± 0.8 and 7.4 ± 4.4 pmole/ml, respectively; Fig. 5c) when its analgesic action is significantly observed. As expected according to its chemical structure and previous half-life evaluation, after 5 h most of LIT-001 was found in urine (188951 ± 8475 pmole/ml; Fig. 5c) and was hardly detectable in both CSF (1.5 ± 1.1 pmole/ml) and brain (0.6 ± 0.4 pmole/ml; Fig. 5c). These results indicate that the long-lasting anti-hyperalgesic effect of i.p. LIT-001 is likely due to its prolonged presence in plasma, with putative central effects.

## Discussion

In the present study, we show that LIT-001, a non-peptidergic specific agonist for OTR, exerts a significant long-lasting (>5 h) anti-hyperalgesic effect on both mechanical and thermal heat sensitivity.

Antinociceptive and analgesic action of OT is well documented^21–23^. It has been shown to act at both peripheric and central levels^10^ mainly through final reduction of spinal wide dynamic range (WDR) neurons and C fiber excitability^11^. However, the main limitations of OT, or OTR peptidergic agonists, are (i) the short duration of the effect, (ii) the lack of permeability through the blood brain barrier (BBB), and (iii) the lack of specificity, which all are not compatible with clinical use.

Here, we show that a low molecular weight, non-peptidergic agonist, LIT-001, exerts a long lasting antihyperalgesic effect, up to 5 h. Two points may explain the prolonged effect of LIT-001. First, it has a long half-life (>2 h), by comparison to OT or OTR peptidergic agonists, which all have a very short half-life of less than 15 min. Second, it may reach its central and/or peripheral targets and trigger here long lasting mechanisms involving OTR. At this point, it is important to highlight that, for clinical purpose and mainly in case of chronic pain, a strong analgesic candidate should not only focus on nociception but also positively modulate all pain-induced disorders, such as anxiety, depression, loss of social interaction, impaired food intake or stress. Indeed, to attenuate this large variety of pain-relative symptoms may significantly improve the patient quality of life, one of the main goals of modern medicine. In this regard, to target the oxytocinergic system, in particular with specific OTR agonist such as LIT-001, may be particularly relevant. Indeed, activation of OTR is known to induce a variety of molecular cascades^24^ resulting in an important modulation of nociception^25^, social recognition and interactions^26,27^, anxiety^28^, feeding behavior^29^, and stress^30^, all important comorbidity factors in painful patients. Interestingly, a previous study has also shown that LIT-001 improved social interaction in a mouse model of autism^20^, reinforcing its putative interest as an analgesic molecule.

Besides, we confirm the capability of LIT-001 to cross the blood brain barrier to exert its action in the central nervous system^20^ (Fig. 5). In addition, we show that LIT-001 exerts long lasting anti-hyperalgesic effects. Importantly, LIT-001 does not accumulate in the body but is almost entirely excreted as such in the urine. While those data were obtained by intra-peritoneal injection, we hypothesize that similar results may be obtained using an oral administration of LIT-001. If true and transposable to human, it may lead to the development of a drug easy to take every few hours to limit pain symptoms. Those aspects point toward LIT-001, and future non-peptidergic agonists, as key candidates for clinical use. Although it displays a low micromolar affinity for this receptor, LIT-001 is a potent V2R agonist in functional assays^20^. The V2 receptor is peripheral and known to regulate water homeostasis. It is thus likely that LIT-001 will have some antidiuretic effect *in vivo* that will have to be studied and taken into account for a potential development toward clinical studies.

Because of its long half-life and its capability to cross the BBB, one may worried about the putative LIT-001 side effects: to design drugs with limited side-effects is a major challenge in chemical and pharmaceutical industries. Importantly, LIT-001 does not interact significantly with any classical off targets (G protein coupled receptors, transporters, enzymes, ion channels listed in Figs. 1, S1), neither as an agonist/activator nor an antagonist/inhibitor. Noteworthy, neither LIT-001 nor the OT antagonist L-368,699 displayed any significant agonist or antagonist activity at the TRPV1 receptor at 5 μM (Fig. 1b). Therefore, LIT-001 is a relatively specific OTR agonist likely exerting its analgesic effect via this receptor. This conclusion is strengthened by our results showing that the LIT-001 antihyperalgesic action is fully prevented in presence of L-368,699, a specific OTR antagonist that is also devoid of significant activity at TRPV1 receptor at 5 μM. In addition, an interesting aspect of LIT-001 seems to be its specific action on nociception and pain. Indeed, while LIT-001 shows limited inhibitory activity of COX2, which in a model of inflammatory pain could be relevant, we observed no modification of the size of the edema induced by CFA injection (Fig. 4), indicating that the LIT-001-induced reduction of pain symptoms is not based on a reduction of inflammation. However, we did not rule specific protocols to tests its anti-inflammatory action *per se*, and this should be done before any clinical trial. In addition, it is important to note that LIT-001 did not modify the mechanical/thermal heat sensitivities in control animals or contralateral paws, indicating that LIT-001 does not have antinociceptive effect, which, in a clinical perspective, is an important characteristic in order to limit undesired loss of sensitivities. At this stage, the only known drawback of LIT-001 is its action at the V2 vasopressin receptor (V2R).

In conclusion, we found that LIT-001 is a very useful probe to validate the oxytocin receptor as a target for the treatment of pain and represents a promising drug-like lead compound for the development of novel treatments.

## Methods

All the protocols, tests and use and living animals were performed in accordance with European committee council Direction, authorization from French Department of Agriculture and from the regional ethic committee (Comité Régional d’Ethique en Matière d’Expérimentation Animale de Strasbourg, CREMEAS).

### Drugs

LIT-001 was prepared as described in Frantz *et al*.^20^. For *in vivo* biological assays, it was dissolved in carboxymethyl cellulose (CMC, 1%) - NaCl (0.9%) and administered at the dose of 10 mg/kg. LIT-001 or vehicle were injected intraperitoneally (i.p.), in a volume of 10 ml/kg^20^, 24 h after the induction of the CFA-induced painful inflammatory sensitization. To confirm the implication of the OTR we injected (i.p.) also L-368,899 (Sigma, St. Louis, MO) (1 mg/kg), an OTR antagonist, in combination with LIT-001 in another group of rats^11^.

### *In vitro* physicochemical and pharmacological characterization

***Solubility of LIT-001*** was determined from solution of about 1 mg of compound in 500 μl of PBS at pH 7.4. (Figs. 1, S1) The solution was stirred at room temperature for 24 h and centrifuged at 15.000 × *g* for 5 min. The supernatant was diluted with a mixture of acetonitrile and water and analyzed by HPLC with a diode detector (Gilson; Kinetex 2.6 μm C18 100 A 50 × 4.6 mm column). No degradation of LIT-001 was observed after 24 h. The lipophilicity (LogD_7.4_) of LIT-001 was measured using 10 ml of a stock solution diluted with different concentrations of octanol and PBS to cover a LogP range from −2 to + 4.5 in a final volume of 1 ml. After stirring for 1 h at room temperature (≈ 20 °C), the samples were centrifuged and the different phases were analyzed by HPLC (Gilson; diode detector, Kinetex 2.6 μ C18 100 A 50 × 4.6 mm column).

***Stability of LIT-001*** was determined in human hepatocytes. Cells were unfrozen and their viability measured (Tryptan blue). Cells are suspended (2 × 106 cells/ml) and dispensed in 96 well microtiterplates (50 μl/well). LIT-001 (50 μl of a 2 μM in the incubation media) was added. The final concentration of the compound was 1 μM in 1 × 10^6^ cells/ml. The incubation volume was 100 μl/well. Incubations were stopped after 0, 4, 20, 40, 80 and 120 min in mixing the well content with 100 μl acetonitrile at 0 °C. A positive control (testosterone) was prepared in same conditions. All samples were analyzed by LC-MS/MS (UHPLC coupled to a triple quadripole Shimadzu LC-MS 8030). Each measurement was performed in triplicate.

***In vitro profiling of LIT-001*** was performed by Eurofins as described in the Eurofins SafetyScreen-Functional panel, 2018^31^. The hepatotoxicity of LIT-001 was studied with cryo-preserved mouse hepatocytes. After unfreezing (optiTHAW and optiINCUBATE media, Xenotech) and cell viability control, cells (2 × 10^6^ cells/ml) were dispatched in 96 well microtiterplates. LIT-001 was added to reach a concentration 1 μM in the presence of 1 × 106 cells/mL in a volume of 100 μl/well. The incubation was stopped at 4, 20, 40, 80 and 120 min in adding 100 μl of acetonitrile at 0 °C. A positive control was treated in the same conditions. All samples were analyzed by UHPLC coupled to a mass spectrometer (Shimadzu LC-MS 8030).

***Cytochrome inhibition by LIT-001*** was studied as follows. A stock solution of LIT-001 at 10 mM in DMSO was prepared and stored at 4 °C. Solutions containing the cytochrome substrates and control inhibitors or LIT-001 were prepared. 2 μl of substrate-inhibitor solutions were mixed with 176 μl of phosphate buffer containing human liver microsomes (0.2 mg/ml), 1 mM of NADPH and 3 mM of MgCl_2_. Height concentrations were tested: 0.03; 0.1; 0.3; 1; 3; 10; 30 and 100 μM. The reaction was initiated by addition of the co-factor after 5 min of incubation at 37 °C. After one hour of incubation, 200 μl of acetonitrile were added to stop enzymatic reactions and solubilize the products. Different control inhibitors were used and supernatants were analyzed by LC-MS/MS (UHPLC separation; Shimadzu LC-MS 8030).

- Furafylline (CYP 1A2 inhibitor): 0.03; 0.1; 0.3; 1; 3; 10; 30 and 100 μM
- Sulfaphenazole (CYP 2C9 inhibitor): 0.03; 0.1; 0.3; 1; 3; 10; 30 and 100 μM
- Tranylcypromine (CYP 2C19 inhibitor): 0.03; 0.1; 0.3; 1; 3; 10; 30 and 100 μM
- Quinidine (CYP 2C19 inhibitor): 0.003; 0.01; 0.1; 0.5; 1; 5; 10 and 50 μM
- Kétoconazole (CYP 3A4 inhibitor): 0.003; 0.01; 0.1; 0.5; 1; 5; 10 and 50 μM

### Animals

Male Wistar rats (300 g; JANVIER LABS, Le Genest St. Isle, France) were used for this study. They were housed by groups of 3 or 4 under standard conditions (room temperature, 22 °C; 12/12 h light/dark cycle) with *ad libitum* access to food and water and behavioral enrichment. All animals were manipulated and habituated to the tests and to the room for at least 2 weeks. All behavioral tests were done during the light period (i.e., between 7:00 and 19:00). All the procedures were performed in accordance with European committee council Direction, authorization from French Department of Agriculture and from the regional ethic committee.

### Behavioral testing

#### Mechanical allodynia

In all experimentations, to test the animal mechanical sensitivity, we used a calibrated forceps (Bioseb, Chaville, France) previously developed in our laboratory (Figs. 2, 3 and S2, S3)^32^. Briefly, the habituated rat is loosely restrained with a towel masking the eyes in order to limit stress by environmental stimulations. The tips of the forceps are placed at each side of the paw and a graduate force is applied. The pressure producing a withdrawal of the paw, or in some rare cases a vocalization of the animal, corresponded to the nociceptive threshold value. This manipulation was performed three times for each hindpaw and the values were averaged averaged.

#### Thermal allodynia/hyperalgesia

To test the animal heat sensitivity, we used the Plantar test with Hargreaves method (Ugo Basile, Comerio, Italy) to compare the response of each hindpaw^33^ when we tested healthy animals (unilateral intraplantar NaCl injection) and animals having received unilateral intraplantar CFA (Freund’s Complete Adjuvant) injection. The habituated rat is placed in a small box and we wait until the animal is calmed then we exposed the hindpaw to a radiant heat, the latency time of paw withdrawal was measured.

#### CFA model of inflammatory pain

In order to induce a peripheral inflammation, 100 μl of complete Freund adjuvant (CFA; Sigma, St. Louis, MO), was injected in the right hindpaw of the rat. All CFA injections were performed under light isoflurane anesthesia (3%). Animals were tested daily for 10 days after the paw injection, a period during which animals exhibited a clear mechanical allodynia and thermal heat hyperalgesia.

### Pharmacokinetics of LIT-001

#### Preparation of brain, cerebrospinal fluid, plasma and urine extracts

Brains from rat injected with 10 mg/kg (18.8 µmol/kg) i.p. of LIT-001 were homogenized with an Ultra Turrax instrument (Ika, Staufen, Germany) in 2 ml of H_2_O (Figs. 4, S4). The homogenates were then sonicated (3 times 10 s, 100 W) with a Vibra Cell apparatus (Sonics, Newtown, U.S.A.). Protein concentrations were determined using the Bradford method (Protein Assay, Bio-Rad, Marne-la-Coquette, France). 400 µl was mixed with 4 ml of ice cold acetonitrile (ACN) and let 30 min on ice. Samples were then centrifuged (20,000 × *g*, 30 min) at 4 °C. Supernatants were dried under vacuum and resuspended in 400 µl ACN 10%/H_2_O 89.9%/formic acid 0.1% (v/v/v) and a volume of 5 µl was injected on the LC-MS/MS. For CSF, plasma and urine, 200 µl of fluids were mixed with 1 ml of ice cold acetonitrile (ACN) and let 30 min on ice. Samples were then centrifuged (20,000 × *g*, 30 min) at 4 °C. Supernatants were dried under vacuum and suspended in 200 µl ACN 10%/H_2_O 89.9%/formic acid 0.1% (v/v/v) and a volume of 5 µl was injected on the LC-MS/MS.

#### LC-MS/MS instrumentation and analytical conditions

Analyses were performed on a Dionex Ultimate 3000 HPLC system (Thermo Scientific, San Jose, USA) coupled with a triple quadrupole Endura mass spectrometer (Thermo Scientific). The system was controlled by Xcalibur v.2.0 software (Thermo Electron). Samples were loaded onto an Accucore C18 RP-MS column (ref 17126–151030; 150 × 1 mm 2.6 μm, Thermo Scientific) heated at 40 °C. The presence of LIT-001 was studied using the multiple reaction monitoring mode (MRM). Elution was performed at a flow rate of 150 µl/min by applying a linear gradient of mobile phases A/B. Mobile phase A corresponded to ACN 1%/H_2_O 98.9%/formic acid 0.1% (v/v/v), whereas mobile phase B was ACN 99.9%/formic acid 0.1% (v/v). The gradient used is detailed in Fig. S4. Electrospray ionization was achieved in the positive mode with the spray voltage set at 3,500 V. Nitrogen was used as the nebulizer gas and the ionization source was heated to 210 °C. Desolvation (nitrogen) sheath gas was set to 27 Arb and Aux gas was set to 9 Arb. The ion transfer tube was heated at 312 °C. Q1 and Q2 resolutions were set at 0.7 FWHM, whereas collision gas (CID, argon) was set to 2 mTorr. Identification of the compounds was based on precursor ion, selective fragment ions and retention times obtained for LIT-001. Selection of the monitored transitions and optimization of collision energy and RF Lens parameters were manually determined (see Fig. S4 for details). Qualification and quantification were performed in MRM mode. Quantification was obtained using Quan Browser software (Thermo Scientific). For tissues and fluids, LIT-001 was quantified using calibration curves of external standards of LIT-001 (125 fmol to 100 pmol/injection; Fig. S4) added to urine, plasma or brain extracts of naive rat and submitted to the same procedure described for respective fluids and tissue recovery. The amounts of LIT-001 measured in samples fit within the standard curve limits, with typical analytical ranges (the range of amounts that can be accurately quantified) from 150 fmol to 120 pmol.

### Statistical analysis

Data are expressed as mean ± standard error of the mean (SEM). Statistical tests were performed with GraphPad Prism 7.05 (GraphPas Software, San Diego, California, USA) using repeated-measures two-way ANOVA, with the following factors: treatment (between), and time (within); when the ANOVA test was significant, the Tukey test was used for *post-hoc* multiple comparisons between individual groups. Results were considered to be statistically significant if p values were below 0.05 (*), 0.01 (**), and 0.001 (***). For the area under the curve (AUC) comparisons, we used the one-way ANOVA (factors: treatment); when the ANOVA test was significant, the Tukey test was used for post hoc multiple comparisons.

## Supplementary information

Supplementary Figures.
