# Supplementary material for: A Nonpeptide Oxytocin Receptor Agonist for a Durable Relief of Inflammatory Pain
Source: Sci Rep. 2020 Feb 20;10:3017. doi: 10.1038/s41598-020-59929-w (PMC7033278; doi:10.1038/s41598-020-59929-w)
Supplement: Supplementary file 1 — Supplementary Figures. [file 41598_2020_59929_MOESM1_ESM.pdf]

A Nonpeptide Oxytocin Receptor Agonist for a Durable Relief of Inflammatory Pain

Louis Hilfiger[1], Qian Zhao[2], Damien Kerspern[1], Perrine Inquimbert[1], Virginie Andry[1], Yannick Goumon[1], Pascal Darbon[1], Marcel Hibert[2#], Alexandre Charlet[1#*]

[1] Centre National de la Recherche Scientifique and University of Strasbourg, UPR3212 Institute of Cellular and Integrative Neurosciences, Strasbourg, France.

[2] Laboratoire d'Innovation Thérapeutique, Faculté de Pharmacie, UMR7200 CNRS/Université de Strasbourg, Illkirch, France.

[#] Equal senior author.

* Corresponding author.

Corresponding Author

Alexandre Charlet, PhD

CNRS UPR3112, INCI

8, Allée du Général Rouvillois

67000 Strasbourg, France

Phone: + 33 (0) 6070 825 06

E-mail: acharlet@unistra.fr

Supplementary table and figures

Channel	Compound	Concentration	Normalized percentage inhibition (mean, n=2)
Nav 1.5	LIT-001	5 mM	0.7
	Lidocaïne	100 mM	78.0
Cav 1.2	LIT-001	5 mM	-9.3
	Verapamil	50 mM	57.5
KCNQ1/minK	LIT-001	5 mM	7.1
	Chromanol 293B	10 mM	51.7
hERG	LIT-001	5 mM	2.1
	Cisapride	0.1 mM	35.9
			Normalized peak current (mean, n=2)
GABA A a1b2g2	LIT-001	5 mM	110.7
	Vehicle	0.33% DMSO	102.6
	Diazepam	0.1 mM	224.8
hnAChR a4b2	LIT-001	5 mM	2.7
	Vehicle	0.33% DMSO	4.5
	Acetylcholine	30 mM	66.1

Figure S1

Figure S1. Inhibition potency of LIT-001 on ion channel blockade. Normalized percentage inhibition values for each compound assayed on each channel specified below. The compound data was normalized to vehicle control (0% inhibition) and maximal inhibition control (100% inhibition).

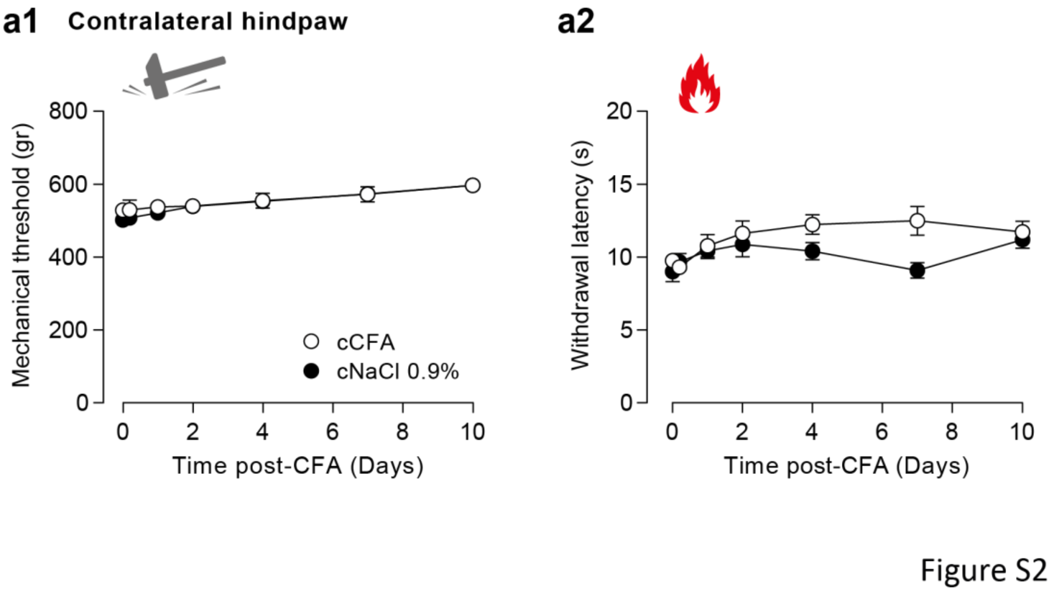

Figure S2. Ten days' time course of long-term modifications induced by CFA subcutaneous injection – contralateral hindpaw. (**a**) Time-course of the CFA-induced mechanical (**a1**) and thermal heat (**a2**) hyperalgesia (CFA, n = 14; NaCl, n = 18). Data are expressed as mean ± SEM. No statistical significance were detected using two-way ANOVA followed by Tukey multiple comparisons test.

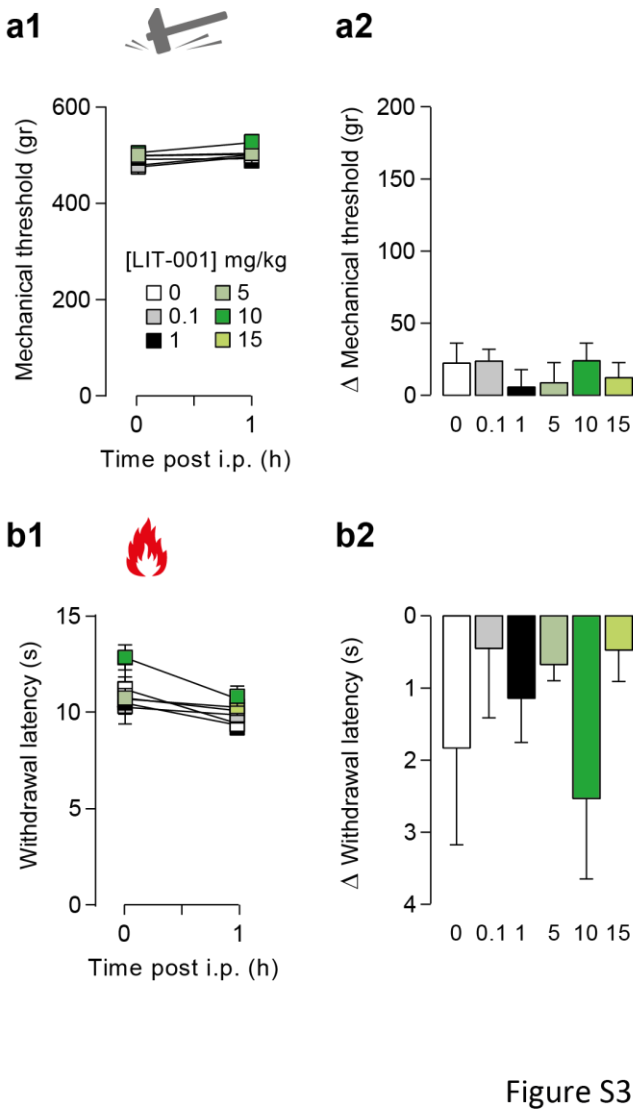

Figure S3. Dose-response of the analgesic properties of LIT-001 on CFA-induced inflammatory pain model – contralateral hindpaw. Effects of LIT-001 0.1 (n = 4), 1 (n = 6), 5 (n = 6), 10 (n = 8) and 15 mg/kg (n = 6) or its vehicule (n = 9) measured 1 hour after i.p. injection on CFA-contralateral hindpaw mechanical (**a**) and thermal heat (**b**) sensitivities. Data are expressed as mean ± SEM.

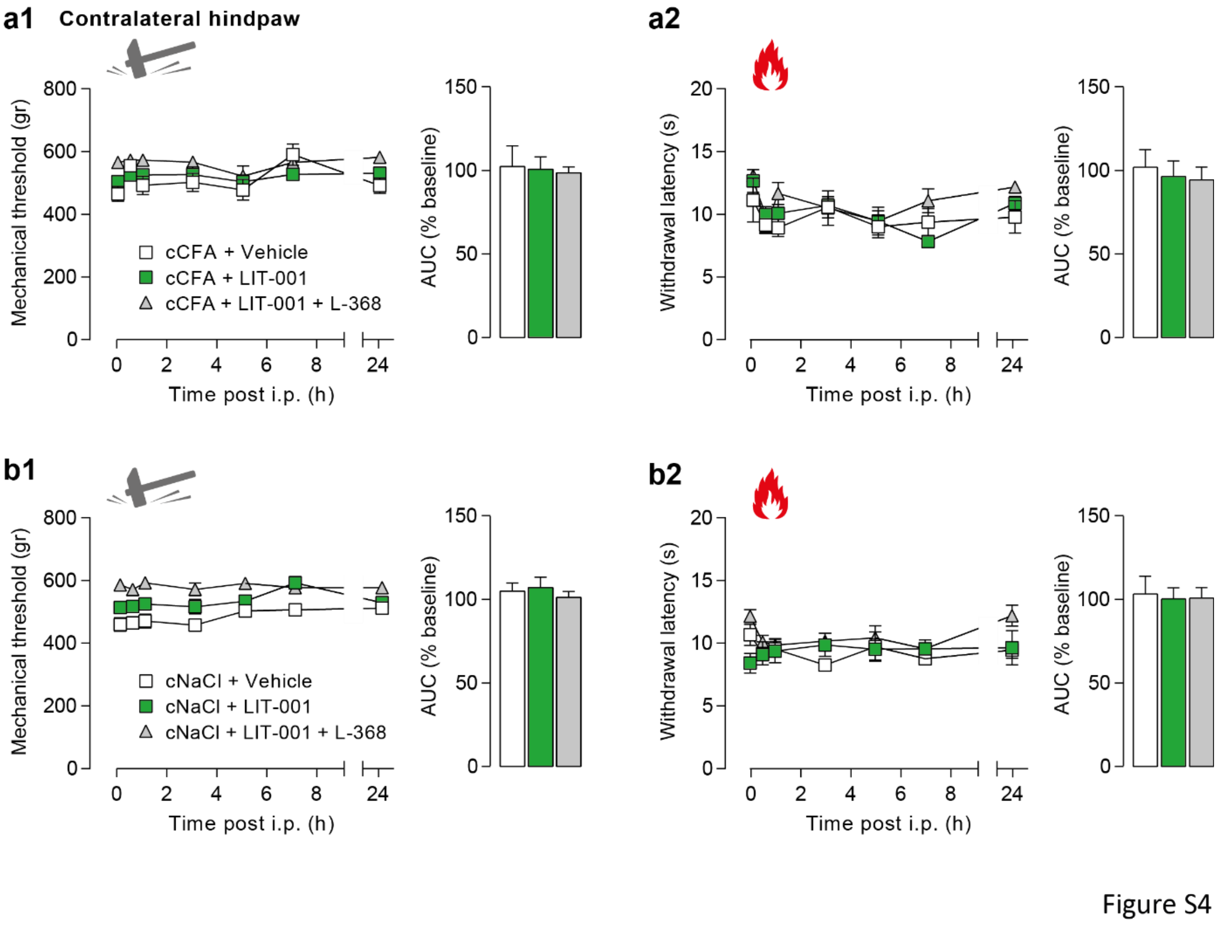

Figure S4. Time-course of the analgesic properties of LIT-001 on CFA-induced inflammatory pain model – contralateral hindpaw. (**a**) Left, time-course of the effects of i.p. LIT-001 10mg/kg (n = 7), its vehicule (n = 5) or co-injection with L-368,699 (n = 6) on CFA-contralateral hindpaw mechanical (**a1**) and thermal heat (**a2**) sensitivities. Right, relative-to-baseline AUC (%) of the effects. (**b**) Left, time-course of the effects of i.p. LIT-001 10mg/kg (n = 7), its vehicule (n = 5) or co-injection with L-368,699 (n = 6) on mechanical (**b1**) and thermal heat (**b2**) sensitivities of NaCl-contralateral hindpaw sensitivities. Right, relative-to-baseline AUC (%) of the effects. Data are expressed as mean ± SEM. No statistical significance were detected using two-way ANOVA followed by Tukey's multiple comparisons test.

a

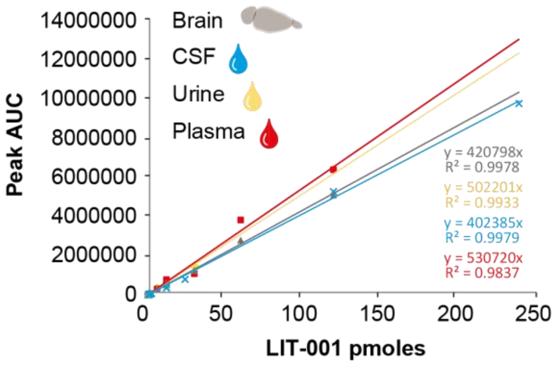

b HPLC gradient

Time (min)	0	3	8	11	14	15	19
B mobile phase	10	10	30	98	98	10	10

c MS ionization, selection, fragmentation and identification parameters

Compound	Polarity	Precursor (m/z)	Product (m/z)	Ion product type	Collision Energy (V)	RF Lens (V)
LIT-001	Positive	532.28	159.11	Quantification	19.05	242.05
LIT-001	Positive	532.28	185.07	Qualification	19.00	242.05
LIT-001	Positive	532.28	332.11	Qualification	22.49	242.05

Figure S5

Figure S5. LC-MS/MS dose response curves of increasing concentration of LIT-001 added to brain extracts, urine, plasma or water. (**a**) Samples were treated as described in the material and method section. (**b-c**) **LC and MS conditions for the purification and the detection of LIT-001.** Mobile phase A corresponded to ACN 1% / H_2O 98.9% / formic acid 0.1% (v/v/v), whereas mobile phase B was ACN 99.9 % / formic acid 0.1% (v/v).
